# Cancer Clusters: Geographic Disparities in Head and Neck Cancer Incidence in Greater Manchester and East Cheshire

**DOI:** 10.7759/cureus.87936

**Published:** 2025-07-14

**Authors:** Nikhil Chotai, Mahesh Yadav, Danuksha K Amarasena, Rohit Kumar

**Affiliations:** 1 Oncology, University Hospitals of Leicester NHS Trust, Leicester, GBR; 2 Otolaryngology - Head and Neck Surgery, The University of Manchester, Manchester, GBR; 3 Diabetes and Endocrinology, The Dudley Group NHS Foundation Trust, Birmingham, GBR; 4 Colorectal Surgery, University Hospitals of North Midlands, Stoke-on-Trent, GBR; 5 Otolaryngology - Head and Neck Surgery, Manchester University NHS Foundation Trust, Manchester, GBR

**Keywords:** cancer clusters, early diagnosis and treatment, epidemiology, geographical disparities, head and neck cancer (hnc), healthcare inequalities, hpv-related cancer, index of multiple deprivation (imd), smoking and alcohol consumption, socioeconomic status

## Abstract

Objective

This study aimed to assess whether a statistically significant correlation exists between socioeconomic status, measured by the Index of Multiple Deprivation (IMD), and the incidence of head and neck cancer (HNC) in Greater Manchester and East Cheshire. Understanding this relationship can help inform targeted public health interventions and reduce healthcare disparities.

Methods

Using Multidisciplinary Team (MDT) lists, 236 HNC patients from Manchester University NHS Foundation Trust were identified. Patient demographics and residential postcodes were extracted from the HIVE electronic patient record system. Postcodes were then mapped to IMD deciles using the UK Government’s official postcode-to-IMD lookup tool. This allowed for the categorisation of patients by area-level socioeconomic deprivation.

Results

The analysis revealed a negative correlation (ρ = -0.21) between socioeconomic status, as indicated by IMD decile, and the incidence of HNC cases. Statistical analysis showed that this correlation was not statistically significant at the α = 0.05 level.

Conclusion

While this study did not identify a statistically significant correlation between socioeconomic deprivation and HNC incidence, a weak negative trend was observed. These exploratory findings suggest potential geographic and socioeconomic patterns that warrant further investigation. Understanding these trends may support more targeted public health strategies - especially in regions with a higher disease burden - and contribute to efforts aimed at earlier diagnosis and improved health outcomes.

## Introduction

Head and neck cancer (HNC) encompasses a spectrum of malignancies affecting various anatomical sites, including the oral cavity, pharynx, hypopharynx, larynx, nasal cavity, and salivary glands. It ranks as the seventh most prevalent cancer globally. The Global Cancer Observatory estimates that approximately 890,000 new cases and 450,000 deaths occur annually due to squamous cell carcinoma (SCC) HNC, constituting about 4.5% of all cancer diagnoses and deaths worldwide [1]. In the United Kingdom alone, approximately 12,200 people are diagnosed with HNC each year, with the majority of these being SCCs [2,3]. HNC is influenced by various risk factors, including smoking, alcohol consumption, human papillomavirus (HPV) infection - particularly HPV 16 - Epstein-Barr Virus (EBV), exposure to radiation, and occupational hazards [4]. 

Extensive research highlights a direct correlation between low socioeconomic status and the risk of HNC, largely due to factors such as smoking [5]. The National Health Service (NHS) is committed to ensuring equal access to high-quality healthcare services for individuals residing in socioeconomically deprived areas [6].

The primary objective of this study was to examine whether a statistically significant relationship exists between area-level socioeconomic deprivation, as measured by IMD deciles, and the standardised incidence rates of HNC within the Greater Manchester and East Cheshire region.

This study holds implications for public health planning and resource allocation, as identifying geographic disparities in HNC incidence can guide targeted interventions aimed at enhancing earlier presentation and diagnosis and access to primary care services. Furthermore, by locating areas with higher incidence rates, our findings can further the development of educational initiatives for raising awareness about the symptoms and risk factors associated with HNC.

We have adopted a retrospective ecological approach, mapping cancer incidence by region, and correlating it with area-level deprivation using national statistics.

Ultimately, this research aims to contribute valuable insights that can support current research, inform decision-making, and optimise healthcare delivery strategies within the Greater Manchester and East Cheshire region.

## Materials and methods

Our study investigated the relationship between socioeconomic deprivation and the incidence of HNC patients by analysing the Index of Multiple Deprivation (IMD) deciles corresponding to patients' residential areas. We hypothesised that there is no statistically significant correlation between the IMD decile and the incidence of HNC.

**Figure 1:**
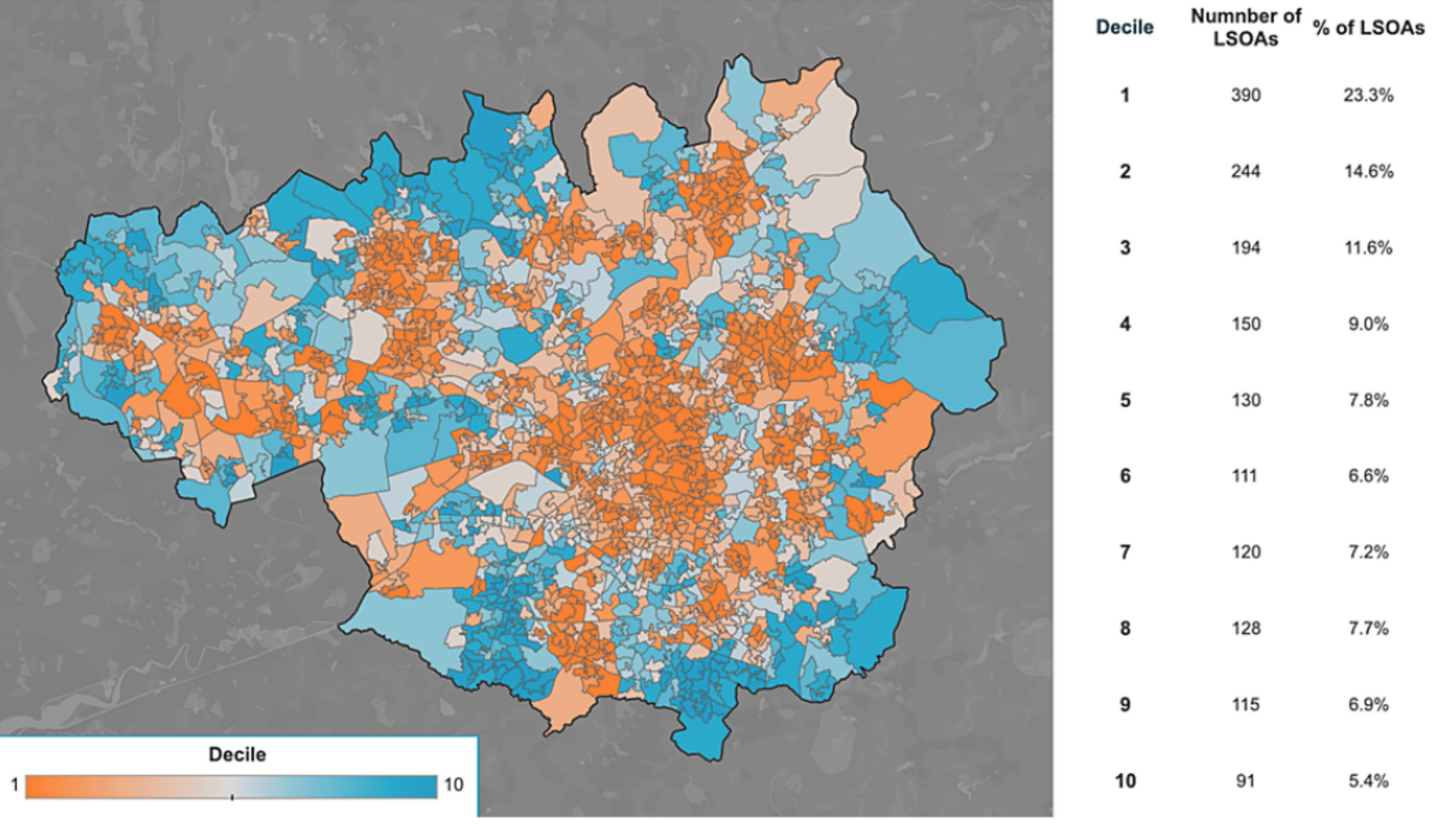
A map to show the distribution of the Index of Multiple Deprivation Deciles within Greater Manchester. Image credit: Image reproduced with permission of the rights holder: Resolve Poverty [6,7]. LSOAs: Lower Layer Super Output Areas

The Indices of Deprivation database in England employs a thorough methodological framework to measure relative deprivation. This database integrates data from seven distinct domains of deprivation, including income, employment, education, health, crime, barriers to housing and services, and living environment. These domains are combined and weighted to calculate the IMD, which divides England into lower-layer super output areas (LSOAs). England comprises 32,844 LSOAs, ranked from 1 (most deprived) to 32,844 (least deprived). These rankings are further grouped into deprivation deciles, as illustrated in Figure 1 and Table 1 [6,7].

**Table 1:** Breakdown of LSOAs into IMD decile. LSOAs: Lower-Layer Super Output Areas; IMD: Index of Multiple Deprivation Table credit: [7]

Decile	LSOAs
1 (most deprived area)	1 to 3,284
2	3,285 to 6,568
3	6,569 to 9,853
4	9,854 to 13,137
5	13,138 to 16,422
6	16,423 to 19,706
7	19,707 to 22,990
8	22,991 to 26,275
9	26,276 to 29,559
10 (least deprived area)	29,560 to 32,844

Data collection

The study focused on patients diagnosed with HNC within the Greater Manchester and East Cheshire area. A retrospective analysis was conducted on all patients listed for HNC management within the Ear, Nose, and Throat (ENT) surgical Multidisciplinary Team (MDT) from January 1, 2024, to March 28, 2024. Data were collected using the HIVE digital patient record system at Manchester University NHS Foundation Trust. Variables collected included patient age, sex, cancer subtype, and full postcode. Data were exported into a spreadsheet format (Microsoft Excel; Microsoft® Corp., Redmond, WA, USA) for preprocessing.

Areas included

Areas were grouped by administrative boundaries (e.g., towns or boroughs), consistent with local government divisions and aligned with available population estimates. The decision to split areas this way was made to reflect natural service catchment boundaries and to facilitate comparison across urban and semi-rural contexts.

The Greater Manchester and East Cheshire region was subdivided into Bolton, Bury, Calderdale, Congleton, Crewe and Nantwich, High Peak, Hyndburn, Lancaster, Macclesfield, Blackburn with Darwen, Manchester, Oldham, Rochdale, Rossendale, Salford, South Staffordshire, St Helens, Stockport, Tameside, Trafford, Wigan, and Wyre.

Patient count

The number of patients diagnosed with HNC was determined for each subset based on the collected data.

IMD decile calculation

IMD deciles were assigned based on each patient’s full postcode using the official government postcode-to-IMD decile mapping service (https://imd-by-postcode.opendatacommunities.org/). For patients living in the same area, IMD scores were averaged to generate a mean decile value for each geographical subset (town/city). The study used the 2019 English Indices of Deprivation dataset. No individual-level deprivation data were used - only area-level scores.

Average IMD decile calculation

The average IMD decile for each subset was computed by summing the IMD decile values for all patients within the subset and then dividing by the total number of patients within that subset.

Population data collection

Population data for each area were obtained from the website (citypopulation.de) [8], which provides estimates of the population within each area as of 2021.

Calculation of HNC incidence per population

The number of HNC patients per population (per 100,000) was calculated for each area using the collected population data. This allowed for the standardisation of HNC incidence rates across different subsets.

Statistical analysis

Statistical analysis was conducted using Microsoft Excel (Office 365, version 2024; Microsoft® Corp.). Spearman’s rank correlation was calculated using the CORREL function on ranked data. The analysis tested the relationship between average IMD decile (predictor) and standardised HNC incidence per 100,000 (outcome) across 22 geographic subsets. Assumptions regarding monotonicity were considered appropriate for this non-parametric test. The resulting correlation coefficient was used to compute a t-statistic and a two-tailed p-value to assess statistical significance at α = 0.05.

## Results

There were 236 patients identified as having a type of HNC from the MDT lists. The majority were males (166, or 70.3%). The mean age of the cohort was 65.24 years (SD = 11.9).

The Spearman’s rank correlation coefficient (ρ = -0.21) was calculated to analyse the relationship between socioeconomic deprivation, as measured by the IMD, and the incidence of HNC across 22 geographic areas. These paired observations are summarised in Table 2, which presents the data for HNC incidence and average IMD decile in each area. To evaluate the strength and significance of the association, a corresponding t-statistic of 0.97 (df = 20) was calculated, yielding a two-tailed p-value of 0.34. This indicates that the observed correlation is not statistically significant at the α = 0.05 level. A summary of the statistical analysis, including the correlation coefficient and significance testing, is provided separately in Table 3.

**Table 2:** Number of HNC patients per 100,000 population in each area, and the corresponding average IMD decile. HNC: Head and Neck Cancer; IMD: Index of Multiple Deprivation

Area	Number of HNC patients per population (per 100,00)	Average IMD decile
Bolton	3.72	4.78
Bury	6.71	6.92
Calderdale	0.48	7.00
Congleton	6.66	8.00
Crewe and Nantwich	1.52	9.00
High Peak	6.60	1.33
Hyndburn	1.22	4.00
Lancaster	1.90	8.00
Macclesfield	14.72	1.38
Blackburn with Darwen	0.65	2.00
Manchester	6.34	3.06
Oldham	14.45	3.69
Rochdale	8.04	4.22
Rossendale	1.41	4.00
Salford	6.30	3.18
South Staffordshire	0.91	9.00
St Helens	0.55	5.00
Stockport	10.52	5.23
Tameside	6.92	3.38
Trafford	8.51	7.20
Wigan	10.63	5.17
Wyre	0.89	6.00

**Table 3:** Results of Spearman’s rank correlation analysis between the average IMD decile and the incidence of HNC per 100,000 population across 22 geographic areas. HNC: Head and Neck Cancer; IMD: Index of Multiple Deprivation

Statistic	Value
Spearman’s p	-0.21
n (pairs of observations)	22
Degrees of freedom (df)	20
t-statistic	0.97
p-value (two-tailed test)	0.34
Statistical significance (α)	0.05
Conclusion	Not significant

**Figure 2:**
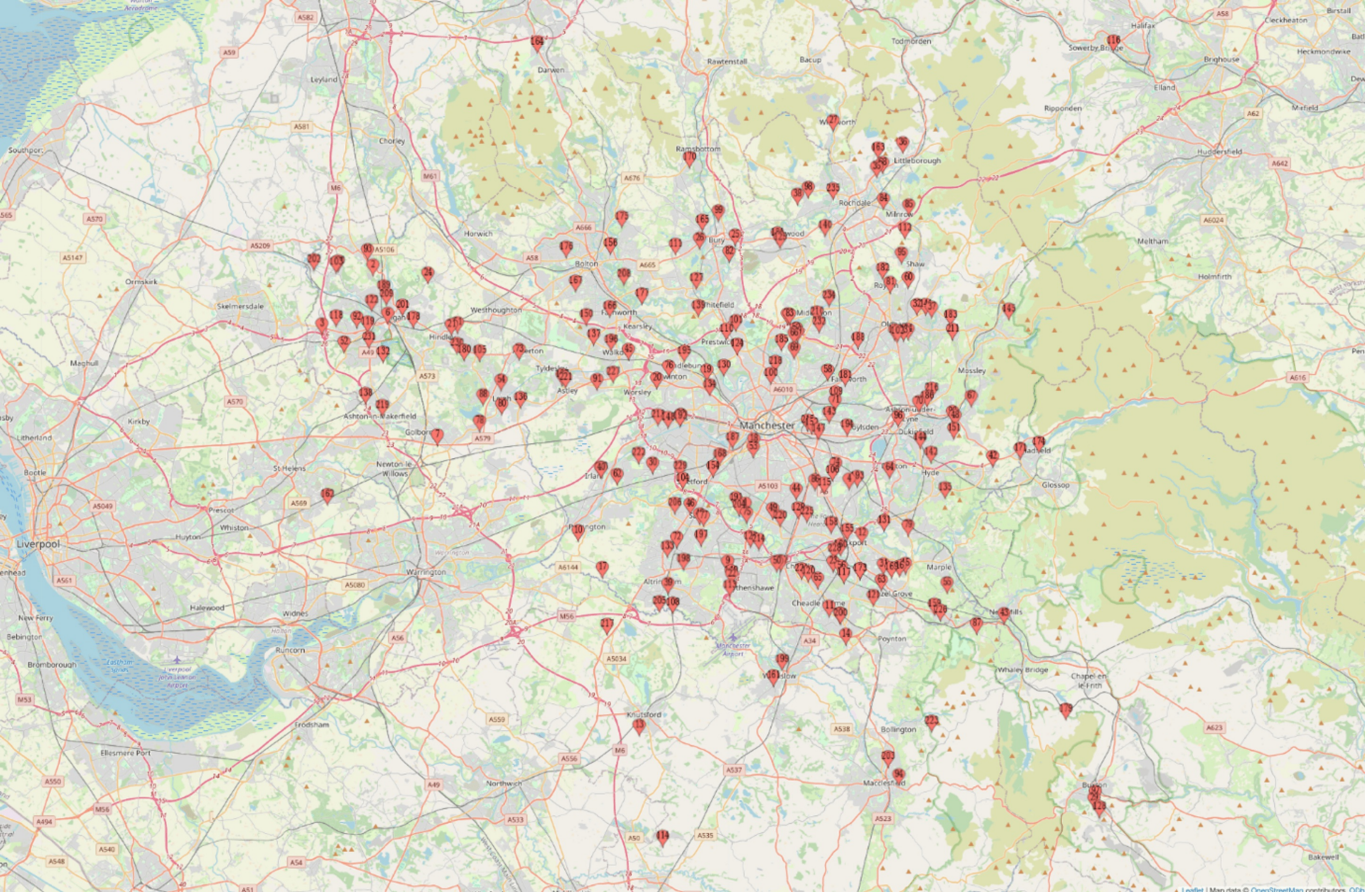
A map showing the geographical distribution of head and neck cancer patients in Greater Manchester. Image credit: The map was created using https://www.mapcustomizer.com/ by the author, Nikhil Chotai.

To visualise the geographic distribution of HNC patients, a map was generated, plotting each patient's location within Greater Manchester and East Cheshire. This allowed for a clear representation of clustering patterns, highlighting areas with higher concentrations of cases (Figure 2).

## Discussion

The Spearman's rank correlation coefficient analysis revealed a weak negative correlation between the incidence of HNC per 100,000 population and the IMD decile. However, this correlation was not statistically significant, and caution is warranted when interpreting this result. One likely factor contributing to the lack of significance is the relatively small sample size and limited number of cases per geographic area, which reduces statistical power (Table 4).

**Table 4:** Number of HNC patients within each area. HNC: Head and Neck Cancer

Area	Number of HNC patients in the study
Bolton	11
Bury	13
Calderdale	1
Congleton	2
Crewe and Nantwich	1
High Peak	6
Hyndburn	1
Lancaster	1
Macclesfield	8
Blackburn with Darwen	1
Manchester	35
Oldham	16
Rochdale	18
Rossendale	1
Salford	17
South Staffordshire	1
St Helens	1
Stockport	31
Tameside	16
Trafford	20
Wigan	35
Wyre	1

This study contributes to the growing body of literature examining the relationship between socioeconomic status and cancer incidence. Prior research has consistently shown that individuals from more deprived backgrounds face a higher risk of developing HNC, largely due to increased rates of smoking and alcohol consumption in these populations [5]. Our findings, while not statistically significant, are consistent with this trend and may reflect underlying behavioural or environmental risk factors associated with deprivation.

The shifting aetiology of HNC further complicates this relationship. While tobacco and alcohol remain dominant risk factors, there has been a rise in HPV-associated oropharyngeal cancers, particularly HPV-16, which are often seen in younger, more affluent patients [9-11]. This evolving landscape may be contributing to the weakening association between socioeconomic status and HNC incidence, especially in regions with declining smoking prevalence.

Methodologically, the use of area-level IMD deciles introduces the potential for ecological fallacy, where assumptions about individual-level socioeconomic status are based on aggregated geographic data. Within-area variability could not be assessed, and individual-level socioeconomic data were not available. Additionally, the use of ENT MDT lists from a single NHS trust to identify patients may introduce selection bias, as some cases managed outside ENT services or at other hospitals may have been missed.

While geographic mapping was employed to visualise the distribution of HNC cases, formal spatial statistical methods were not used. As such, any apparent clustering patterns should be interpreted cautiously. Future research, using spatial statistical approaches (e.g., Moran’s I or spatial regression), could validate and build upon these observations.

**Figure 3:**
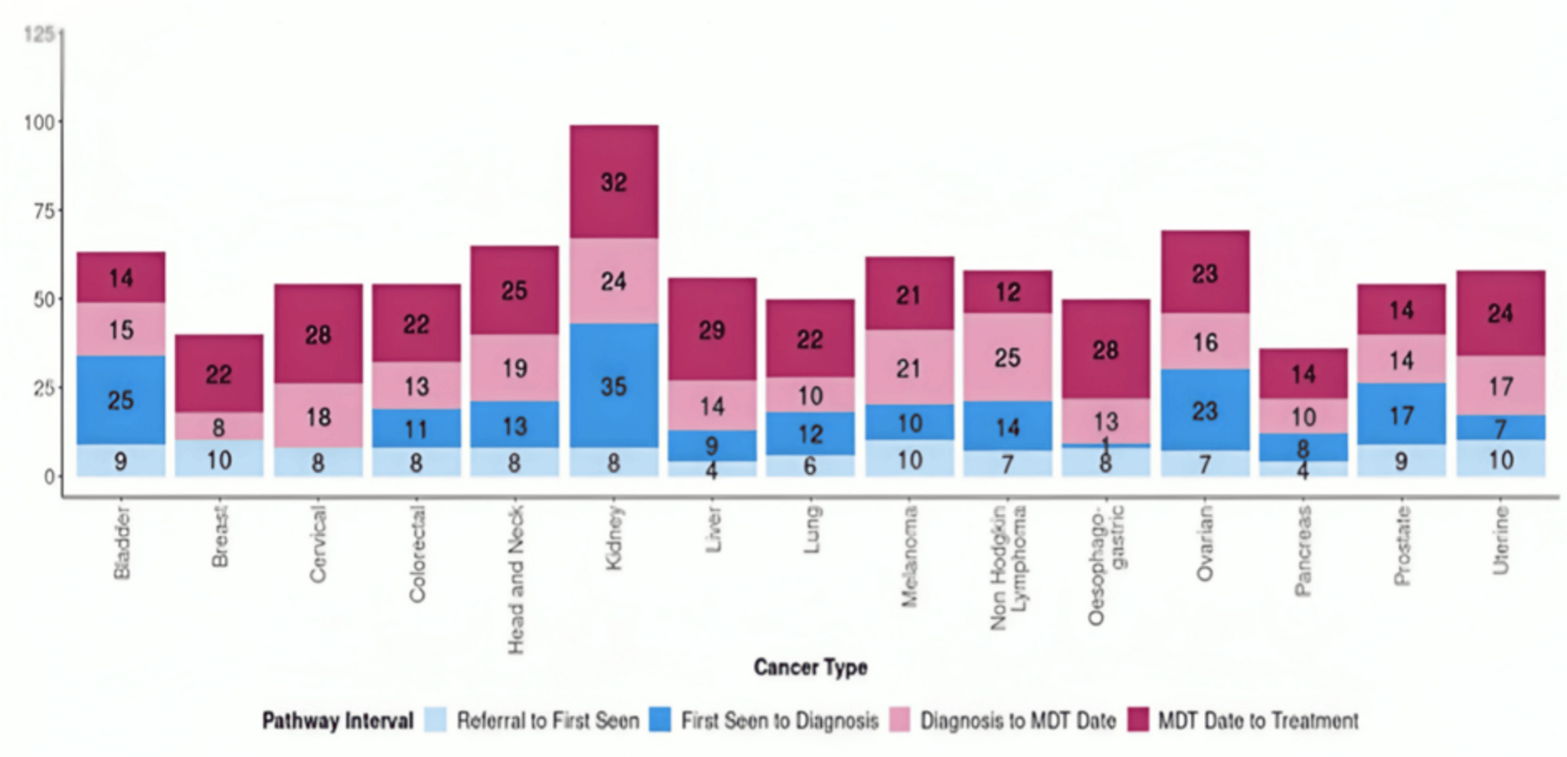
Median days from referral to treatment by cancer type, England (2018). Image credit: [12] This information is licensed under the Open Government Licence v3.0. To view this licence, visit https://www.nationalarchives.gov.uk/doc/open-government-licence MDT: Multidisciplinary Team

Delays in diagnosis and treatment initiation remain a significant concern for HNC patients in the UK. Failure to meet national diagnostic targets - such as the 62-day referral-to-treatment guideline - can allow tumours to progress and lead to poorer prognoses. NHS data from 2018 to 2020 show that only 61% of patients with HNC began treatment within the recommended timeframe, among the lowest for all cancers (Figure 3) [12]. These findings highlight the need for targeted interventions in regions with higher incidence to ensure earlier presentation, timely referral, and access to treatment.

Improving outcomes for HNC patients will likely require both patient-level and system-level approaches. Enhancing public awareness of early symptoms may encourage more timely presentation to primary care, while improving referral practices and education among GPs and other frontline clinicians could expedite diagnosis and treatment initiation. The geographic disparities identified in this study suggest that such interventions could be strategically targeted to areas with higher incidence, supporting more equitable access to care, and improved cancer outcomes.

Limitations

This study focused on patients diagnosed with HNC within the Greater Manchester and East Cheshire region, which may limit the generalisability of findings to other geographical areas. The sample size of 236 patients, collected over a three-month period, may not be sufficient to fully capture the diversity of socioeconomic and demographic factors influencing cancer incidence. In addition, population estimates were derived from 2021 data, which may not accurately reflect the population size during the study period.

Socioeconomic status was approximated using area-level IMD deciles linked to patient postcodes. This approach carries a risk of ecological fallacy, where area-level deprivation is assumed to reflect individual socioeconomic circumstances, which may not always be accurate. Furthermore, patients were identified through ENT MDT lists from Manchester University NHS Foundation Trust, which may not include all patients within the region, particularly those treated outside ENT services or at other trusts, introducing potential selection bias.

Data collection and IMD mapping involved manual steps, such as postcode matching and spreadsheet handling, which could introduce human error in geolocation or categorisation. Statistical analysis was performed using Microsoft Excel; while appropriate for preliminary analysis, future studies may benefit from the use of dedicated statistical software (e.g., SPSS or R) to enhance robustness and reproducibility. Lastly, while spatial visualisation was used to highlight regions with high case concentrations, formal spatial statistical methods were not applied, and therefore, any observed “clustering” should be interpreted with caution.

## Conclusions

In conclusion, this study found a weak, non-significant negative correlation between socioeconomic deprivation and HNC incidence across Greater Manchester and East Cheshire. While the findings are not statistically conclusive, they highlight potential geographic and socioeconomic patterns in cancer distribution that warrant further investigation.

Given the limitations of sample size, regional focus, and the use of area-level deprivation data, these findings should be regarded as hypothesis-generating rather than definitive. Future research should leverage larger, population-wide datasets, incorporate individual-level socioeconomic information, and apply formal spatial statistical methods to strengthen the evidence base. Such efforts will be critical for informing equitable healthcare planning, targeting early detection initiatives, and addressing persistent disparities in cancer outcomes.
